# Factors associated with timely initiation of antenatal care among pregnant women in Bahir Dar city, Northwest Ethiopia: Cross‐sectional study

**DOI:** 10.1002/nop2.1162

**Published:** 2021-12-23

**Authors:** Azimeraw Arega Tesfu, Amlaku Mulat Aweke, Getahun Belay Gela, Kihinetu Gelaye Wudineh, Fentahun Yenealem Beyene

**Affiliations:** ^1^ Department of Midwifery College of Medicine and Health Sciences Bahir Dar University Bahir Dar Ethiopia

**Keywords:** antenatal care, Ethiopia, pregnant, timely initiation, women

## Abstract

**Aim:**

This study aimed to assess the timely initiation of Antenatal Care and associated factors among pregnant women attending antenatal care clinics at Bahir Dar city, North West Ethiopia.

**Design:**

Institutional based cross‐sectional study was conducted.

**Methods:**

Data were collected on 804 pregnant women from 20 February to 27 March 2017. Face‐to‐face interview through systematic sampling technique was applied. Binary logistic regression was performed using SPSS software version 21, and the level of significance of association was determined at *p*‐value <0.05 with a 95%confidence interval.

**Results:**

This study identified 44.2% of pregnant women started their first antenatal care timely. Maternal secondary and above level of education AOR = 7.07 (95% CI: 4.41, 11.35)), age at first pregnancy >18 years AOR = 2.77 (95% CI: 1.39, 5.57) and having information about the correct time of ANC booking AOR = 3.14 (95% CI: 1.67, 5.92) were significantly associated with timely commencement to first antenatal care.

## 1 INTRODUCTION

Despite several efforts were made to reveres maternal mortality, it is still a global public health problem. Globally, maternal mortality rate is 216 per 100,000 live births with a large proportion of deaths due to early preventable or treatable pregnancy and childbirth complications, 99% of maternal deaths occur in low‐ and middle‐income countries, in sub‐Saharan African countries alone accounting for 66% (Alkema et al., 2016; World Health Organization (WHO) 2016a, 2016bWorld Health Organization). Ethiopia is one of the Sub‐Saharan African countries with the highest maternal mortality ratio (MMR) which is 412 maternal deaths per 100,000 live births (Central Statistical Agency (CSA) [Ethiopia] and ICF, 2016).

Antenatal care (ANC) is one of the key strategies designed to minimize such devastating conditions through providing health promotion, prevention, detection and treatment care for pregnancy‐related illnesses (World Health Organization (WHO), 2015). Moreover, ANC can reduce both maternal and neonatal mortality by detecting at‐risk pregnancy and initiating appropriate medical and educational interventions towards the risk associated (Federal Ministry of Health (FMOH) [Ethiopia], 2010; World Health Organization (WHO), 2015; World Health Organization (WHO) 2016a, 2016b).

Therefore, timely ANC initiation is crucial for better woman's pregnancy experience and the successes of maternal and neonatal health by early identifying, managing and preventing any problems through supplementation of micronutrients like iron and folic acid, screening of infection (e.g. HIV, Syphilis and hepatitis) and health promotion (Furness et al., 2011; Prathapan et al., 2011; Roth et al., 2011; Ӧzge et al., 2017; World Health Organization (WHO) 2016a, 2016b).

Many maternal and prenatal deaths occur in women who have not received timely, inadequate and no utilization of ANC (Oyerinde, 2013). Early antenatal care attendance during the first three months of gestation plays a major role in detecting and treating complications that occur during pregnancy (Abou‐Zahr and Wardlaw, 2003).

According to the World Health Organization (WHO) Focus antenatal care model recommendation, all pregnant ladies are better to start their first antenatal care (ANC) booking within the first trimester of pregnancy (8–12 weeks) (World Health Organization (WHO) (2002)).

The global existing evidence displays that the prevalence of early booking of antenatal care visits was 43%. There is a high inconsistency between regions, which showed that in developed 85%, in developing below 45% and in sub‐Sahara region less than 25% (Moller et al., 2017). Despite focussed ANC is provided for free in Ethiopia, according to the Ethiopian demographic health survey (EDHS) 2016 report, only 20% of women received their first ANC during the first trimester of pregnancy(Central Statistical Agency (CSA) [Ethiopia] and ICF, 2016). Different literature conducted in Ethiopia also revealed that the prevalence of early booking of ANC visits ranges from 13.2% to 65.6% (Amtatachew et al., 2013; Belayneh et al., 2014; Ewnetu et al., 2015; Feleke et al., 2015; Tolera Gudissa et al., 2015; Yilal, 2015).

Studies conducted in developing countries showed that age of the mother, residence, educational status of the mother, occupation, age at first marriage, age at first pregnancy, parity, planned or wanted pregnancy, information access, advised booking within 12weeks and decision‐making power were factors for early ANC booking (Adekanle & Isawumi, 2008; Ajayi & Osakine, 2013; Amtatachew et al., 2013; Banda et al., 2012; Belayneh et al., 2014; Erica, 2012; Ewnetu et al., 2015; Feleke et al., 2015; Ochako & Girchuhi, 2016; Tolera Gudissa et al., 2015; Yilal, 2015). This study aims to assess the prevalence and factors associated with early antenatal care initiation of mothers in health facilities.

### 1.1 Study setting

This study was conducted at Bahir Dar City public health institutions, Bahir Dar, North West Ethiopia. Bahir Dar City is the capital city of the Amhara National Regional State, located Northwest at about 565 km far from the capital city of Ethiopia (Addis Ababa). In the city, there are Ten Governmental health centres, one regional referral hospital, One District Hospital owned by the government. In the city, there are 17 urban administrative kebeles and 4 special towns. Based on the 2007 National Census conducted by the Central Statistical Agency of Ethiopia (CSA) projection, the town has a total population of 848,596.

## 2 METHODS

### 2.1 Study design and period

An institutional‐based cross‐sectional study was carried out in Bahir Dar City public health institutions from 20 February to 27 March 2017, North West Ethiopia.

#### 2.1.1 Study participants

All pregnant mothers who visited public ANC clinics were included in the study, whereas pregnant mothers who were seriously ill and in labour pain were excluded.

In this study, timely visit of first antenatal care is visiting ANC service before and at 12 complete gestational weeks (World Health Organization (WHO) 2016a, 2016b).

### 2.2 Sample size determination and techniques

The sample size for the study was calculated by using single population proportion formula with the following assumptions. By assuming a margin of error of 5%, 95% CI (1.96), proportion =47.7% (Belayneh et al., 2014), non‐response rate of 5% and by considering design effect 2, the final sample size was found to be 804.

Generally, a multi‐stage sampling technique was used. Then, seven governmental health institutions were selected out of 12 governmental health institutions randomly by lottery method. The average daily client flow receiving the service in the selected health institutions was taken from the registry book. According to the expected number of women in the specified period of data collection, the sample size was proportionally allocated to those health institutions. Finally, the study participants were selected by using a systematic sampling technique.

### 2.3 Data collection tools and procedures

The questioner was developed by authors after reviewing different kinds of literature on the topic and validated by professional experts. Data were collected using structured and pre‐tested interviewer‐administered questionnaires through face‐to‐face exit interviews, which consists of a sociodemographic characteristic, obstetric information and other related variables. The data on the timing of the first ANC booking were gathered from women's recall, if the failure to remember, and it was extracted from their medical charts. The questionnaire was first prepared in English and then translated to Amharic (local language) and back to English again by a language expert to maintain consistency. Seven qualified diploma midwife data collectors were selected from other catchments which are not selected for data collection and two midwives having masters' in clinical midwifery were recruited to supervise the data collection process.

### 2.4 Data quality control

Two consecutive days of training to the data collectors and supervisors on the objectives, relevance of the study, ethical concern and techniques of interviews were given before the actual data collection. Before data collection, Research Ethics Committee approval was taken from the IRB of Bahir Dar University, College of Medicine and health sciences, and informed consent and confidentiality were assured by data collectors to the participants. The Amharic version of the questionnaire was pre‐tested on 40 (5%) pregnant mothers of the sample population in the health institutions not included in the study, and then, the instrument was amended accordingly. The data collection is strictly followed and supervised daily by supervisors along with the principal investigators. Data coding and entry were checked throughout the work. Data cleaning was checked at the end of the data entry.

### 2.5 Data processing and analysis

All the questionnaires were checked visually, and data cleaning, coding and entry were done by using EPI Info version 7.0 and exported to SPSS version 21.0 software package for analysis. Descriptive and summary statistics like frequency, percentage, mean and standard deviation were carried out. Tables and graphs were also used for data presentation. Bivariate and multivariable logistic regression analyses were used to identify variables associated with timely antenatal care initiation. Variables with a *p*‐value ≤0.20 in the bivariate logistic regression analysis were selected into the multivariable logistic regression model for controlling the possible effect of confounders. Finally, variables that had an independent association with timely antenatal care initiation were identified based on the adjusted odds ratio (AOR), with 95%CI and *p*‐value less than 0.05. Ethical clearance was obtained from the institutional review board of Bahir Dar University. A formal letter request of cooperation was written to the Bahir Dar city health office. Written consent was obtained from each study participant. Confidentiality of information and privacy was maintained.

## 3 RESULTS

### 3.1 Socio‐demographic characteristics of respondents

All 804 pregnant women were participated making a response rate of 100%. Among the total participants, 82% of the respondents were in the age group of 20‐35years. The mean age of the respondents was 26.3 (*SD* ±4.9 years). Less than half (47.3%) of participants' educational level was secondary and above. Three hundred and sixty‐three (45.1%) of the respondent travelled 30–60 min to reach the ANC services (Table 1).

**TABLE 1:** Socio‐demographic characteristics of pregnant women in Bahir Dar city, North West Ethiopia, 2017(*n* = 804)

Variables	Categories	Frequency	Per cent
Age in years	<20	102	12.7
	20–34	659	82
	≥35	43	5.3
Residence	Urban	619	77
	Rural	185	23
Ethnic	Amhara	793	98.6
	Others^a^	11	1.4
Religion	Orthodox	734	91.3
	Muslim	62	7.7
	Others^a^, ^b^, ^c^	8	1.0
Marital status	Married	778	96.8
	Single	20	2.5
	Others^c^	6	0.7
Educational status	No formal education	278	34.6
	Primary school (1–8)	146	18.1
	Secondary and above	380	47.3
Occupation	Employed	163	20.3
	Self Employed	54	6.7
	Housewife	523	65
	Daily labourer	64	8.0
Distance in minutes	≤30	266	33.1
	30–60	363	45.1
	>60	175	21.8

^a^ Oromo, Tigre.

^b^ Catholic, Protestant.

^c^ Divorced, Widowed.

### 3.2 Obstetric characteristics of respondents

Among married women, 450 (57.4%) of respondents' age at first marriage was ≥18 years. More than two‐thirds of their age at first pregnancy was above eighteen years. Fifty hundred twenty‐five (65.7%), of respondents, had parity one and above. The majority (75.5%) of respondents perceived that the correct time of first ANC booking is within 12 weeks of gestation (Table 2).

**TABLE 2:** Obstetric characteristics of pregnant women in Bahir Dar city, North West Ethiopia, 2017(*n* = 804)

Variables	Categories	Frequency	Per cent
Age at first marriage(*N* = 784)	<18	334	42.6
	≥18	450	57.4
Age at first pregnancy	≤18	160	19.9
	>18	644	80.1
Parity	Nulliparous	279	34.3
	Para one and above	525	65.7
Planned or wanted pregnancy	Yes	658	81.8
	No	146	18.2
Having information about ANC initiation time	Yes	651	81
	No	153	19
Decision‐making power on ANC initiation time	Yes	757	94.2
	No	47	5.8
Mothers who perceived the right time to start ANC	≤12 week	607	75.5
	>12 week	197	24.5
Timing of first ANC booking	0–12 week	355	44.2
	13−27week	415	51.6
	28−42week	34	4.2

### 3.3 Timing of initial antenatal care visit

**FIGURE 1:**
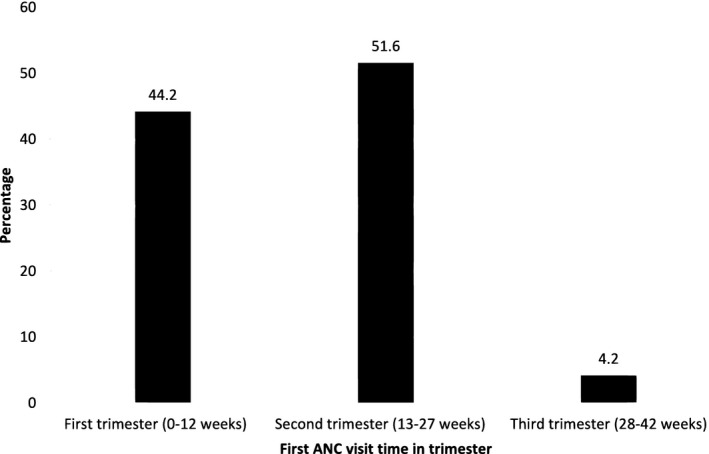
Timing of initial antenatal care visit

This study identified that 44.2% with 95%CI (40.6, 47.8) of respondents were booking their first ANC in a timely within 12weeks of gestation. The rest study participants initiated the first ANC. The mean gestational age of starting the first ANC visit was 15.9 (*SD *± 6.03 weeks). The timing of the first ANC booking was ranging from 4 weeks up to 36 weeks of gestational age (Figure 1).

### 3.4 Reasons for late antenatal care initiation

**FIGURE 2:**
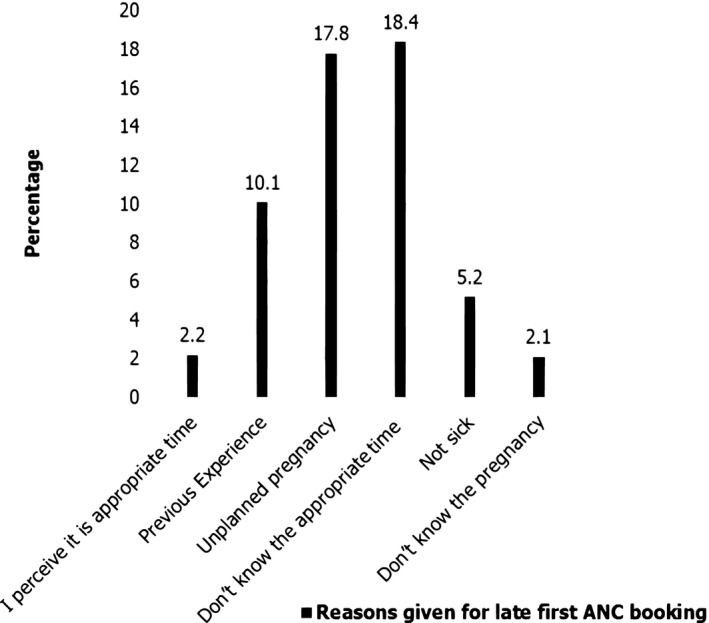
Reasons for late antenatal care initiation

Among respondents who started their first ANC booking late, 18.4% of them reported that do not know the appropriate time, and 2.2% of respondents perceived it as the appropriate time (Figure 2).

### 3.5 Factors associated with the timely visit of first antenatal care

Bivariate analysis showed that residence, women educational status, partner educational status, women occupation, age at first pregnancy, having information about the correct time of ANC booking, mothers who perceived the right time to start ANC, having decision‐making power to use ANC and for whom mothers inform their pregnancy at the first time were a candidate variables for multivariable analysis at p‐value less than 0.2. In multivariable analysis, women with a secondary and above level of education (AOR: 7.07, 95% CI: 4.41, 11.35), women's age (>18 years) at first pregnancy (AOR: 2.79, 95% CI: 1.39, 5.57) and having information about the correct time of ANC booking (AOR: 3.14, 95% CI:1.67, 5.92) were significantly associated with timely first ANC booking at *p*‐value less than 0.05 (Table 3).

**TABLE 3:** Association of factors with a timely booking of first ANC in Bahir Dar city, North West, Ethiopia

Variables	Time at first ANC visit				COR (95%CI)			AOR (95%CI)
	Booked timely		Booked late					
Residence								
Urban	333		286		8.62 (5.38,13.84)			
Rural	22		163		1			
Women educational status								
Secondary and above	278		102		12.28 (8.78,17.18)			7.07 (4.41, 11.35) **
Primary and bellow	77		347		1			**1**
Partner educational status								
Secondary and above	279		127			22.22 (12.94, 38.16)		
Primary (1–8)	55		134			4.15 (2.31, 7.48)		
Unable read and write	17		172			1		
Occupation								
Employed	140		77			12.72 (5.77, 28.08)		
Housewife	207		316			4.58 (2.14, 9.82)		
Daily labour	8		56			1		
Age at first marriage								
>18 years	338		306			9.29 (5.49, 15.72)		2.77 (1.39, 5.57)*
≤18 years	17		143			1		1
Having information about ANC initiation time								
Yes	332		319			5.88 (3.68, 9.41)		3.14 (1.67, 5.92)**
No	23		130			1		1
Mothers who perceived the right time to start ANC								
≤12 week	279		328				1.35 (1.02, 1.88)	
>12 week	76		121				1	
Decision‐making power to use ANC								
No	8		39				1	
Yes	347		410				0.24 (0.11, 0.53)	
For whom Mothers inform the pregnancy at the first time								
To husband	266	372		1.37 (0.69, 2.74)				
Her Mother	76	52		2.81 (1.32, 5.99)				
Others^a^	13	25		**1**				

*p‐value < 0.005, **p‐value < 0.001.

^a^ Sister and friend.

## 4 DISCUSSION

A large proportion of maternal death worldwide was due to early preventable or treatable pregnancy and childbirth complications (Alkema et al., 2016; World Health Organization (WHO) 2016a, 2016b). Ethiopia is one of the Sub‐Saharan African countries with the highest maternal mortality ratio (Central Statistical Agency (CSA) [Ethiopia] and ICF, 2016). Antenatal care help to ensure the well‐being of the mother and foetus through early detection of risks in pregnancy, prevention of pregnancy and labour complications and ensures the safe delivery of mother and child (Federal Ministry of Health (FMOH) [Ethiopia], 2010; World Health Organization (WHO), 2015; World Health Organization (WHO) 2016a, 2016b).

This study identified only about 44.2% of respondents have started their first ANC booking within the recommended time with 95% CI (40.6, 47.8). This result showed that ANC in the study area did not meet the WHO antenatal care model recommendation, which states that every pregnant woman should start the first ANC visit within the first trimester of pregnancy (World Health Organization (WHO) (2002)). This finding was in line with the finding of previous studies conducted in Gondar, Ethiopia 47.4% (Belayneh et al., 2014). However, the finding of this study was lower than the study conduct in Addis Ababa, Ethiopia 65.6% (Yilal, 2015). This might be since Addis Ababa is the capital city of Ethiopia, women's level of awareness could be better than the study area. Moreover, the possible explanation for this difference, the community in Addis Ababa might have better accessible and available health services than the study area.

On the contrary, the finding in this study was higher when compared with studies from the different areas of African countries, Zimbabwe less than 1% (Erica, 2012) and Zambia 30% (Banda et al., 2012). The possible explanation for this variation might be the presence of socio‐economic differences and socio‐cultural status variations, time gap, availability and accessibility of health services. The finding of this study was higher than the national finding Ethiopian DHS 20% (Central Statistical Agency (CSA) [Ethiopia] and ICF, 2016). The explanation might be due to the time gap. Furthermore, this might be due to a difference in the scope of the study, to the fact that the national study covered more rural areas, and this study was conducted at an institution; therefore, women were more likely to attend ANC earlier than the general population.

Similarly, this finding was also higher than a study done in a different area of Ethiopia, in Ambo town 13.2% (Tolera Gudissa et al., 2015), Arba Minch Town 17.4% (Feleke et al., 2015), Jimma University Specialized Hospital 39.9% (Ewnetu et al., 2015) and Debre Brhan 26.2% (Amtatachew et al., 2013). This inconsistency could be attributed to the time gap, accessibility of health services and improvement in health service delivery. Moreover, the finding might be also justified by the fact that due to the efforts done by the Ethiopian government health policy to strengthen strategies for maternal and child health morbidity and mortality reduction through increasing utilization of maternal health services, particularly increasing ANC utilization (Federal Democratic Republic of Ethiopia [FDRE] & Ministry of Health [MoH], 2015).

Factors that positively affect the timely ANC booking were women's secondary and above level of education, age at first pregnancy and having information about ANC initiation time.

Women with a secondary and above level of education were 7.07 times more likely to be booked earlier than their counterparts. This finding agrees with the studies done in Kenya (Ochako & Girchuhi, 2016), south Nigeria (Adekanle & Isawumi, 2008), Tanzania (Ajayi & Osakine, 2013) and in Ethiopia Gondar (Belayneh et al., 2014), Addis Ababa (Yilal, 2015). The possible explanation could be due to women with a secondary and above level of education might be more likely to be employed, financially independent, and knowledgeable on the necessity of having antenatal care follow‐up during pregnancy, as a result, they might be more likely to realize the importance of early booking their first ANC visit timely. Evidence suggests that educated and knowledgeable mothers are more likely to initiate their first ANC timely (Hagey, 2014).

On the other hand, pregnant mothers who were above eighteen years at the time of first pregnancy were near 2.77 times more likely to be booked their ANC visit within the first trimester of pregnancy than younger women. There is a limited finding concerning the relationship between the timing of first ANC booking and age at their first pregnancy in the previous study, but in this study, age at their first pregnancy with above eighteen years was one of the determinant factors for the timing of initiation of ANC. The explanation could be the fact that awareness and level of decision‐making increases with age and those teenagers might feel shame by their pregnancy.

Additional statistical analysis of this study showed that having information about the correct time of ANC booking was a factor for an early ANC visit. Respondents who had information about the time of ANC initiation were 3.14 times more likely to book their first ANC visit within the recommended time compared to respondents who were not informed. This finding was in line with a previous study conducted in Addis Ababa and Gondar (Belayneh et al., 2014; Yilal, 2015). It might be explained by exposure to information on early initiation of ANC visit, enhances women's level of understanding and increases knowledge on the benefit of timely booking. Therefore, this suggested that proper information and advice on a pattern of ANC utilization is important to book at the recommended time.

## 5 CONCLUSION

Even though the ANC services are available free to all women, the timely visit of the first ANC was still low in the study area. Maternal education, age at first pregnancy and having information about the correct time of ANC booking were significantly associated with the timely booking of first antenatal care. Thus, encouraging women's education, avoiding teenage pregnancy and creating awareness for the initiation of the first ANC booking within the recommended time are highly advised.

### 5.1 Limitation

- This study was not triangulated with the qualitative method.

## CONFLICT OF INTEREST

I attest that this manuscript is the authors' original work and has not been submitted to, nor is under review at, elsewhere and is prepared following the instructions to authors' guideline. All authors have contributed to this manuscript, reviewed and approved the current form of the manuscript to be submitted.

## AUTHOR CONTRIBUTIONS

Fentahun Yenealem Byene, Amlaku Mulat Aweke and Getahun Belay Gela inception designed the protocol, data analysis and interpretation. Azimeraw Arega Tesfu and Kihinetu Gelaye Wudineh participate in the data collection, the editorial, and data entry and analysis. The manuscript was drafted by Azimeraw Arega. Finally, the manuscript was revised and approved by all authors.

## ETHICS APPROVAL AND CONSENT TO PARTICIPATE

Ethical clearance was obtained from the institutional review board of Bahir Dar University. A formal letter request of cooperation was written to the Bahir Dar city health office. Written consent was obtained from each study participant. Confidentiality of information and privacy was maintained.

## CONSENT FOR PUBLICATION

Not applicable.

## Data Availability

The data that support the findings of this study are available from the corresponding author upon reasonable request.
